# Schrödinger's Doctrine of Identity: On the Role of Advaita Vedānta in Erwin Schrödinger's Thought

**DOI:** 10.1002/bewi.202400027

**Published:** 2025-06-26

**Authors:** Thijs M. K. Latten

**Affiliations:** ^1^ Department of Values, Technology and Innovation Faculty of Technology, Policy and Management Delft University of Technology

**Keywords:** biography, Erwin Schrödinger, Indian philosophy, metaphysics, philosophy of science

## Abstract

Ever since Erwin Schrödinger learned about Indian thought through Arthur Schopenhauer, it occupied a visible role in both his published writings and personal books. Schrödinger called for a “blood transfusion” of Indian thought into the West and, in one notebook, construed the Upaniṣadic slogan “Brahman = Atman” as the “closest thing to the truth.” However, the historical and philosophical literature on his engagement with Indian ideas remains limited and often confused. Two questions should be addressed for a more comprehensive account of Schrödinger's philosophical views: which Indian insights did he embrace, and what was their role in his thought? I argue that examining what he termed the Indian “doctrine of identity” illuminates answers to these questions and can correct some historical misinterpretations. First, situating Schrödinger's reading of Indian works in his time and analyzing his personal notebooks reveals the dominance of Śaṅkara's Advaita Vedānta reading of the Upaniṣads. Second, by analyzing Schrödinger's published writings and personal notebooks, I argue that this doctrine of identity offered Schrödinger religious consolation, but, furthermore, that Schrödinger took these Indian ideas seriously in his philosophy as well. I highlight how Schrödinger adopted this doctrine of identity into his metaphysical ruminations about the nature of reality and show how it resonates with some of his reflections in the philosophy of science.

## 1 Introduction

Although Erwin Schrödinger is famous for his contributions to quantum physics, he wrote in his book *My View of the World* about a long‐cherished wish to become a philosopher—indicating he considered to “devote” himself to philosophy in 1918, before his 1925 breakthrough in wave mechanics.^1^ This same year, Schrödinger read the work of various philosophers and was introduced to Indian philosophy through Arthur Schopenhauer's writings. In the rest of Schrödinger's writings throughout his life, both published and unpublished, Indian concepts and ideas played a visible role. Most notably, his book *My View of the World* culminated in a chapter called “The Vedantic Vision,”^2^ where he elaborated his answer to what he saw as the “real difficulty” for philosophy, namely: how to make sense of the spatial and temporal multiplicity of observing and thinking individuals.^3^ His answer: “The plurality that we perceive is only *an appearance; it is not real*.”^4^ Schrödinger laid out that ultimately there is a unity of individual selves and a unity between this self and the world:

[…] inconceivable as it seems to ordinary reason, you—and all other conscious beings as such—are all in all. Hence this life of yours which you are living is not merely a piece of the entire existence but is in a certain sense the *whole*; […]. This, as we know, is what the Brahmins express in that sacred, mystic formula which is yet really so simple and so clear: *Tat tvam asi*, this is you. Or, again, in such words as ‘I am in the east and in the west, I am below and above, *I am this whole world*.’^5^

Schrödinger described this insight of ultimate unity as the “profound rightness of the basic conviction of Vedanta”^6^—essentially captured in what he called the “doctrine of identity.”^7^

The main aim of this paper is to zoom in on Schrödinger's doctrine of identity to assess the relation between Indian philosophy and Schrödinger's thought in a twofold manner: I aim to assess 1) which Indian insights Schrödinger specifically embraced; and 2) what role these Indian ideas played in his life and thought.

A more comprehensive understanding of Indian insights in the worldview of this pivotal physicist is particularly relevant in light of Schrödinger's own comments on the matter. Schrödinger adamantly believed that metaphysics was and should be the basis for science,^8^ that this basis was defunct,^9^ and that it was “Eastern thought” that should aid in the rethinking of this basis.^10^ To better understand what Schrödinger could have meant and potentially to better understand the role of Schrödinger's philosophical views in his physics, we first need to reach a better understanding of Schrödinger's thought in general, specifically about the origin and role of the Indian ideas in it.

Previous literature on Schrödinger and Indian thought remains rather limited and often confused. In Lisa Wessels’ 1975 dissertation on Schrödinger's interpretation of quantum mechanics, she centrally assessed his philosophical views through Ernst Mach and Ludwig Boltzmann.^11^ In 1989, Walter Moore published his biography on Schrödinger, addressing Schrödinger's fascination with Indian philosophy more thoroughly. Moore emphasized how Schrödinger adopted Indian concepts as religious truths^12^ and indicated, furthermore, that Schrödinger “found in the philosophy of Vedānta his understanding of the nature of scientific research,”^13^ but Moore did little to elaborate on how exactly this is the case. In 1998, Michel Bitbol then delivered the most philosophically rich account of Schrödinger and Indian thought to date, discussing in quite some detail Schrödinger's thoughts on the duality of mind and matter and the observed plurality of minds and bodies.^14^ Bitbol concluded Schrödinger's philosophy “relied extensively on Indian concepts of mind and matter”^15^ and that “Schrödinger was so deeply immersed in a non‐dualist Vedântic‐like view that this served as a broad framework, and as a subliminal inspiration, in all parts of his work, including in theoretical physics.”^16^

A point of confusion throughout this literature resides in the Indian traditions from which Schrödinger distilled his doctrine of identity. Both Wessels and Moore equated the message of the Upaniṣads (widely regarded as one of the most important foundational collections of Sanskrit texts based on which numerous Indian philosophical traditions arose) with Vedānta (one particular school of interpretations of, among others, the Upaniṣadic texts).^17^ Furthermore, Bitbol referred to Schrödinger's position as Vedānta,^18^ but then also referred to Schrödinger's position as Advaita Vedānta (a further subschool within the school of Vedānta), and makes no attempt to address the historical confusion. The first aim of this paper, discussed in Section 2, is to target this confusion surrounding the origin of Schrödinger's doctrine of identity. I show it is specifically the notion of Advaita Vedānta by eighth century CE Indian thinker Śrī Śaṅkarācārya (hereafter referred to as Śaṅkara) that Schrödinger embraced in his reading of the Upaniṣads and consequently in his doctrine of identity. By historically contextualizing Schrödinger's reading of Indian thought—currently absent from the literature—through nineteenth‐century philosopher Arthur Schopenhauer, we can see exactly why it is this Advaita Vedānta reading that has been embraced in various Western references to the Upaniṣads.

The second and final aim of this paper is to indicate the role of these Advaita Vedānta insights in Schrödinger's life and thought, as discussed in Section 3 and 4. This second aim of the paper contributes to the literature by expanding upon Bitbol's work and by offering a new look into some of Schrödinger's notebooks in Section 3.1. I argue, in this paper, that Schrödinger's published and unpublished writings indicate that this doctrine of identity seems to have offered Schrödinger both a way of dealing with the finitude of existence while also informing his views on the nature of reality and some of his reflections in the philosophy of science. I indicate the philosophical significance of two notions present in the doctrine of identity in Schrödinger's thought (the notion of non‐dualism and of a perceived‐ultimate distinction). I use these notions to analyze the relation between Schrödinger's embrace of this doctrine of identity and Schrödinger's reading of Mach's neutral monism and highlight resemblances with his thoughts on the subject–object relation in quantum mechanics^19^ and his views on scientific realism.

Some notes on methodology and scope are in order. In this paper, I investigate both Schrödinger's published philosophical books and essays as well as his unpublished personal notebooks and diaries (cited as this paper progresses). I closely read selected parts of the Erwin Schrödinger Archive in Vienna relevant to the topic of this paper—selected through database searches and complemented by selections of Christof Capellaro (administrator of the Erwin Schrödinger Archive). Concerning the scope of this paper, I cannot aim to do justice to all philosophers and physicists who have exerted an influence on Schrödinger's thought.^20^ I specifically zoom in on those influences necessary to understand Schrödinger's doctrine of identity, most notably nineteenth‐century philosopher Arthur Schopenhauer and eighth‐century Indian thinker Śaṅkara. One can observe possible parallels particularly with the topics discussed and insights presented by Immanuel Kant and Ernst Mach. Although I will address some of these, a comprehensive study of the relation between these European ideas and possible resemblances in India is beyond the scope of this paper. How exactly the deeper relations between these influences converge in Schrödinger's thought is a topic for future studies—this paper aims to contribute to first laying out what Indian insights Schrödinger so visibly embraced, where they came from, and what role they played in his thinking.

## 2 Śaṅkara and Schrödinger's Reading of the Upaniṣads

### 2.1 Schopenhauer and Persistent Misconceptions

Schrödinger's notebooks around 1918, near the end of the First World War, display a preoccupation with Indian philosophy.^21^ In Schrödinger's own recollection, he became familiar with the “doctrine of unity” (usually referred to as “doctrine of identity”)—which he attributes to the Upaniṣads—in 1918 through Arthur Schopenhauer.^22^ This is not surprising, as Schopenhauer is one of the most famous (or infamous) and widely read philosophers who introduced Indian thought to the West. Although he did not specify his source, Walter Moore noted multiple times that Schrödinger “read every word” of Schopenhauer,^23^ and concluded Schopenhauer's “direct influence on Schrödinger was considerable, but equally important was the introduction he provided to Indian philosophy.”^24^ This informs an essential but currently overlooked way in which Schrödinger's reading of Indian thought should be contextualized.

Schopenhauer read the Upaniṣads—widely regarded as one of the most important foundational collections of Sanskrit texts in orthodox Indian traditions—through the first translation of the work into Latin, translated by Abraham Hyacinthe Anquetil‐Duperron in 1801.^25^ Schopenhauer reportedly read this book every night before going to bed.^26^ Anquetil‐Duperron's translation of the Upaniṣads, however, was no clear rendering of the Sanskrit text into Latin. Rather, Duperron translated the work based on the Persian translations, dated 1657, commissioned by Prince Dārā Shukōh (great‐grandson of the Mogul emperor Akbar). As historian Wilhelm Halbfass showed, within this Persian translation, various cases of commentaries can be found that can be traced back to eighth‐century Indian thinker Śaṅkara.^27^ This particular reading, as I will illustrate, seems to have informed Anquetil‐Duperron, Schopenhauer, and eventually Schrödinger's readings of the Upaniṣadic texts.

I should first address why rendering Śaṅkara's commentary as a clear reading of the Upaniṣads is historically problematic. This becomes clear once we understand the lineage of Indian scholarship. Vedic texts (or: Vedas)—a large body of early Sanskrit texts—date back to the second millennium BCE. The Upaniṣads is the collection of texts that (partly) complete the Vedas and are dated around the eighth century BCE. The Upaniṣads centrally address various questions (regarding, e.g., the nature of the self, or the epistemology of liberation) and contain verses on key concepts in Indian philosophy, such as *ātman* (the self), *brahman* (ultimate reality), karma, *saṃsāra* (worldly existence), *mokṣa* (enlightenment), *puruṣa* (persons) and *prakṛti* (nature), which come to constitute the vocabulary of later traditions. Various philosophical schools arose that accepted the authority of the Vedas in the following centuries, such as Vaiśeṣika, Nyāya, Mīmāṃsā, Vedānta, Sāṃkhya, and Yoga. As the number of interpretative schools suggests, the meaning and significance of the terms and topics discussed in (among others) the Upaniṣads, are widely controversial throughout later traditions. These traditions developed distinct epistemologies, metaphysics, ethics, soteriology, and logical frameworks. To give an example of the variety already within the metaphysical outlook, Vaiśeṣika traditions can be characterized as pluralistic realism where the ontology is organized in categories (*padārtha*), while Samkhya traditions chiefly emphasize the significance of the metaphysical duality between *prakṛti* (nature) and *puruṣa* (persons). The school of Vedānta—often mentioned by Schrödinger and scholars of Schrödinger—places particular emphasis on those passages concerning the relation between *brahman* and *ātman*. Moreover, the school of Vedānta,^28^ as one of the six major interpretive schools of the Vedas listed above, is itself not a unitary school. It can be subdivided into the six main interpretive schools of Bhedābheda, Advaita, Viśiṣṭādvaita, Dvaita, Śuddhādvaita, and more recently Neo‐Advaita—all with a distinct focus. While the subschool of Dvaita Vedānta, for example, teaches the complete difference between the individual self and *brahman*, Advaita Vedānta emphasizes the non‐dualism, or rather, complete monism, of *brahman* and *ātman* (this can be recognized in the name of the school: *a* meaning non and *dvaita* meaning dualism in Sanskrit). As the Indian philosophical traditions are much too rich and diverse to do justice to here, what is important to observe in the context of this paper is that Śaṅkara and his school of Advaita Vedānta is distinctly promoting a radical form of metaphysical monism.^29^ *Brahman*, for Advaitins, is the monist unity that transcends individuality and plurality. Śaṅkara's Advaita Vedāntin commentary, the reading included in Anquetil‐Duperron's translation, thus delivered a specific eighth‐century CE interpretation, within a distinct school of interpretations, on the foundational texts of the Upaniṣads—sixteen centuries (!) after the Upaniṣads came into existence. Hence it would be historically problematic to unequivocally render this reading as a clear reading of the original texts. What Schopenhauer understands to be the Upaniṣads is, thus, influenced by an interpretation (Śaṅkara's reading) within a school of interpretations (Advaita Vedānta) that sits within a further distinct school of interpretations (the school of Vedānta) of the original Upaniṣads.

This intermingling of Śaṅkara's Advaita Vedānta and the original Upaniṣads has affected this line of scholarship philosophically—from the earliest Persian translations into Schopenhauer. Halbfass elaborated that Dārā Shukōh (who commissioned the translations of the Upaniṣads including some of Śaṅkara's commentary into Persian) took it for granted that Hinduism is monotheistic, and saw the Upaniṣads as the “most original testimony of the oneness of God or the Absolute”^30^—resonating with the philosophical tenets of ultimate monism that identified Śaṅkara's Advaita Vedānta in the Indian philosophical landscape. Resemblances between Schopenhauer and Śaṅkara's systems of thought have often been remarked.^31^ Perhaps most importantly, Śaṅkara's notion of the illusory character of the plurality we perceive around us (as ultimately there is only *brahman*) is present in both Anquetil‐Duperron and Schopenhauer.^32^ In a hermeneutical study on Schopenhauer's philosophy and Indian thought, Douglas Berger showed that *māyā* is one of the central terms Schopenhauer used in his philosophical system that he adopted from Indian thought, affecting Schopenhauer's epistemology, metaphysics, and ethics.^33^ Berger showed Schopenhauer incorporated a notion of *māyā* (as ignorance) in his “falsification thesis”^34^—the existence of the world as it presents itself to us is falsified in the sense that it is illusory—construing this notion of *māyā* as a “veil of deception.”^35^ Berger showed, furthermore, that the definition of *māyā* Schopenhauer used is one that was not introduced in Indian traditions until Advaita Vedānta thinkers such as Śaṅkara altered the conception of *māyā* by equating *māyā* with *avidyā* (ignorance).^36^ So, it was this understanding of *māyā* introduced by Advaita Vedānta scholars that influenced Schopenhauer's philosophy.

Despite widespread criticism of Schopenhauer's grasp of Indian philosophy, spanning various misappropriations and generalizations of Indian concepts,^37^ there have been numerous scholars who have accepted Schopenhauer as an authority on Indian philosophy and who have consequently adopted much of Schopenhauer's representations of it—including adopting this reading influenced by Śaṅkara's Advaita Vedānta comments as a faithful reading of the original Upaniṣads. Berger identified two camps of scholars: those who critique Schopenhauer's appropriations and those who naïvely assume the correctness of Schopenhauer's musings on Indian philosophy.^38^ This second, naïve camp includes philosopher Friedrich Nietzsche and Indologist (and Nietzsche's classmate) Paul Deussen.

### 2.2 Schrödinger's Reading of Indian Thought

Now, turning to Schrödinger, there are both historical and philosophical reasons that indicate he might be placed in what Berger called the naïve camp regarding the misconceptions present in Schopenhauer's rendering of Indian thought, both in his first encounters with Indian thought and in the later stages of his life. Let us start with Schrödinger's initial engagement with Indian thought, particularly assessing the sources Schrödinger embraced in his early reading of Indian philosophy.

Although Schrödinger was critical of Schopenhauer as a person,^39^ he nowhere adopted a critical attitude to Schopenhauer's use or understanding of Indian thought—neither in his published writings nor in his personal notebooks. Schrödinger listed in one of his notebooks the sources he read on various Indian traditions. Although this notebook is not dated, there is strong reason to believe it was written around 1918.^40^ Interestingly, the list shows Schrödinger read the work of Max Müller (in original English), who was, according to Berger, critical of Anquetil‐Duperron's translations of the Upaniṣads (the translations Schopenhauer used).^41^ However, the work where Müller expresses this criticism of Anquetil‐Duperron is not referred to by Schrödinger.^42^ Moreover, while Schrödinger listed various authors on Buddhism, and Müller is mentioned in the list of “general” Indian thought, Schrödinger mentions only Paul Deussen's *Das System des Vedânta* when it comes to the sources he read on “Brahmanism.”^43^ In his encounters with Indian thought around 1918, Schrödinger thus appeared to be, in his first reading of Vedāntic traditions, particularly exposed to the work of Deussen, a notoriously naïve scholar regarding Schopenhauer's misconceptions. Deussen is known to have privileged Śaṅkara's Advaita Vedānta reading, which is particularly evident in the book by Deussen that Schrödinger quoted to have read around 1918.^44^

Assessing Schrödinger's philosophical ruminations in these early years of his readings of Indian thought confirms the suspicion of Schrödinger as naïvely regarding Schopenhauer's musings as accurate. In the 1925 parts of *My View of the World*, an Advaita Vedānta notion of *māyā*, where *māyā* is this “veil of deception” that Schopenhauer and Deussen adopted, underlies Schrödinger's reasoning when he writes about the fundamental dogma of “Vedantic philosophy” as the idea that the plurality we perceive is only an appearance.^45^ But even more importantly, Schrödinger's doctrine of identity, which entailed radical unity of all individuals and a further unity between this universal self and the world, centrally formulated in 1925 as Schrödinger's answer to the “real difficulty” for philosophy,^46^ precisely captured the central idea Śaṅkara's Advaita Vedānta aimed to convey. Although unity was a theme present in the original Upaniṣadic texts, it was precisely the Advaita Vedānta school (including Śaṅkara), as elaborated earlier, that centrally emphasized the illusory character of perceived appearances and embraced the idea of ultimate non‐duality, that is, unity, of *ātman* and *brahman*. Śaṅkara and his school of thought distinctly underscored this idea as *the* message of the Upaniṣads—just like Schrödinger centrally did in his formulation of the doctrine of identity. Schrödinger called this emphasis on non‐plurality the “orthodox dogma of the Upanishads” in 1925, displaying he adopted an Advaita Vedānta reading.^47^ The main idea Schrödinger takes away is Śaṅkara's emphasis, just like Schopenhauer and Deussen.

A natural question to ask is whether Schrödinger was exposed to or searched for other sources that may have been critical of Schopenhauer's presentations at a later stage of his life, for example, when Schrödinger moved to Berlin in 1927 or to Dublin in 1938. Unfortunately, not a lot is known about exactly what sources on Indian thought were available to Schrödinger throughout his life. Schrödinger often wrote on Indian philosophy in his published works and in his personal notebooks but only sporadically referred to the interpreters or authors he read on the matter. To my knowledge, the only name Schrödinger refers to later in his life regarding his reading of Vedāntic traditions is that of Deussen. In the year before his death, in the 1960 part of *My View of the World*, Schrödinger wrote about the doctrine of identity and referred to two works of Deussen as the “best sources available in German,” and Schrödinger continues in the book to quote from Deussen's translations.^48^ In one of these works that Schrödinger labeled the best works on Indian thought, several parts of Deussen's translation of the Upaniṣads were based on Anquetil‐Duperron's Latin translation, which, as elaborated earlier, was precisely the work that intermingled Śaṅkara's commentaries with the original texts (based on the Persian translations of Dārā Shukōh).^49^ Hence Schrödinger appeared to rely on translations by Deussen, while Deussen utilized the very same work responsible for the prevalence of Śaṅkara in Schopenhauer.^50^ As such, despite the lack of comprehensive insight into what sources were available to Schrödinger at different stages of his life, Schrödinger's strong endorsement of Deussen's work near the end of his life in 1960, and Deussen being known for privileging Śaṅkara's reading and adopting Schopenhauer's misunderstandings, displays historical reasons suggesting a certain continuity of the prevalence of Śaṅkara's thought in Schrödinger's views on Indian philosophy.

This suggested continuity is again supported when we look at the content of Schrödinger's reflections on Indian thought in the later stages of his life. Schrödinger wrote explicitly about the Indian concept of *māyā*, again through Schopenhauer's and Śaṅkara's use of the term as the perceived deception (e.g., 1944 and 1960). For example, Schrödinger wrote in 1960: “the plurality of sensitive beings is mere appearance (maya); in reality they are all only aspects of the one being.”^51^ Moreover, the doctrine of identity is possibly even more fanatically embraced in the later stages of his life and centrally echoes the same Advaita Vedānta emphasis on unity throughout his writings (e.g., in 1944, 1958, and 1960).^52^ For example, Schrödinger wrote in 1960: “‘There is no plurality here whatever.’ This is simply the mystical‐metaphysical doctrine of the Upanishads itself […].”^53^ Schrödinger perceiving this doctrine of identity as the “doctrine of the Upanishads itself” in 1960, illustrates he, like Schopenhauer and Deussen, followed Śaṅkara's reading of the work, supposedly until the end of his life.^54^ Even in case Schrödinger had at some point encountered other sources on Indian philosophy that did not adopt Schopenhauer's and Deussen's presentations, the aforementioned historical and philosophical reflections suggest such sources may not have made a significant impact on the central idea of Schrödinger's doctrine of identity.

We see, thus, how Schopenhauer's use of Anquetil‐Duperron's translations resulted in an overemphasis on Śaṅkara's Advaita Vedānta in Western literature on India^55^—which became particularly visible in Schrödinger's doctrine of identity. This should be addressed as there seems to be a way in which we can speak of a lineage of misconception that perpetuates until today, running through Schopenhauer, Deussen, Schrödinger, and now through Schrödinger scholars such as Wessels and Moore. Outside of Bitbol^56^—who correctly referred to Schrödinger's reading of the Upaniṣads as an Advaita Vedānta reading but did not elaborate on why this was the case, nor did he address the historical misconceptions—the conceptual mix‐up originating in Anquetil‐Duperron's 1801 translation remained unnoticed in Schrödinger scholarship. Schrödinger scholars such as Lisa Wessels accepted the “one mind thesis” as the message of the Upaniṣads^57^ (while this emphasis on *brahman* as the metaphysical unity being equal to the universal self, or universal consciousness, is specifically an Advaita position). Furthermore, Schrödinger's biographer Walter Moore understands *māyā* as the “principle of illusion,”^58^ signaling Moore also adopted the Advaita Vedānta definition of *māyā*. The problem is sketched most tellingly when Moore wrote that Śaṅkara “provides a standard and orthodox view of Vedānta,” which is simply untrue, and then continued to elaborate on the message of the Upaniṣads by quoting Śaṅkara.^59^ Such remarks not only perpetuate misconceptions but amplify and solidify them—contributing to a widespread misunderstanding of Indian philosophical traditions in the West.

### 2.3 Critiquing the Substantiality of the Personal Self

Before I discuss this doctrine of identity in Schrödinger's life and thought, I should briefly underline the centrality of this doctrine of identity in Schrödinger's use of Indian thought. Although Schrödinger read widely into Indian thought, reading and writing also about Samkhya, Yoga and Buddhism,^60^ the role of these other schools—except for Buddhism^61^—seems relatively limited in the philosophical work they did in his writings. In this section, I briefly comment on Schrödinger's conceptualization of the self in a notebook in 1944, where I argue Schrödinger's comments can be understood through Śaṅkara's writings. My conclusion contrasts that of Michel Bitbol, who argues that Schrödinger incorporated the Buddhist notion of *anātman* in his conceptualization of the self.

**Figure 1:**
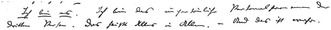
Excerpt from Schrödinger's diary dated April 24, 1944.^68^ Erwin Schrödinger, Diary March−July 1944 [Ephemerides], 1944, Austrian Central Library for Physics, Vienna/Erwin Schrödinger Archive, L33‐557, copy, in parts transcribed by Auguste Dick.

In a diary entry from April 24, 1944, Schrödinger wrote that the ego is transitory, seemingly aligning his ideas with the defining Buddhist tenet of *anātman* (no‐self).^62^ However, in the 1944 diary entry, Schrödinger tried to elaborate that the definition of the self has two meanings: one that is immediate but accidental^63^ and one in which the self is equal to “It,” elaborating this second position by stating that “I am It. […] That is called all in all—and that is true” (**Figure** **1**).^64^ Śaṅkara wrote in the *Brahmasūtrabhāṣya*: “The individual soul is not directly the highest Atman […] We may call the jīva [personal self] as a mere reflection of the Atman.”^65^ This verse (as well as others) is interpreted as arguing that the personal self is a combination of reality and appearance—it is real in so far as *ātman* is its basis, and it is appearance in so far as it is observed as finite and distinct.^66^ Schrödinger's postulation of the two definitions of the self in his diary resonates with Śaṅkara's description: Schrödinger's accidental definition resonates with Śaṅkara's appearance (it appears to us as distinct and finite, but is only appearance), and Schrödinger's definition of the I as the “It,” as “all in all” resonates with Śaṅkara's ultimate definition (Śaṅkara's *ātman,* since *ātman* is equal to the ultimate monist unity for Śaṅkara). Schrödinger, furthermore, often explicitly calls this ultimate reality (this second definition) “Atman.”^67^

Although Buddhist schools would agree with the illusory nature of the accidental ego/personal self, they would not agree with the idea that there would be a substantial *ātman* underneath this illusory self: the rejection of the substantial *ātman* is the primary reason Buddhists reject the authority of the Vedas. Conceptualizing Schrödinger's comments on the self through an Advaita framework allows us to make sense of his two definitions of the self in his diary without running into complications with Buddhist thought rejecting the substantiality of the universal self, which Schrödinger explicitly embraced.

Bitbol concluded Schrödinger incorporated the Buddhist concept of *anātman* into his Advaita Vedānta perspective, resulting in a philosophy that combined Buddhist critiques of the substantiality of the personal self with an Advaita emphasis on the universal self.^69^ I argue this is not a necessary conclusion. One *could* thus construe the transitory and non‐ultimate character of the self as a form of *anātman* in Schrödinger's comments, as Bitbol did, but only in a very limited sense: this only accounts for one of the two definitions Schrödinger and Śaṅkara have for the self and would thus (in the Buddhist notion of *anātman*) be inconsistent with the broader metaphysical claims that both Schrödinger and Śaṅkara put forth. I do not dismiss the role of Buddhism in Schrödinger's thinking (including in how he construes the personal self). For example, reading Buddhist works may reasonably have enforced Schrödinger's belief in the transitory and non‐ultimate character of the personal self. I merely argue, contrary to Bitbol, that the Buddhist notion of *anātman* is not necessary to make sense of Schrödinger's criticism of the substantiality of the personal self, and thereby highlight a way in which we can construe Schrödinger's comments on the personal self through his over‐arching conviction of the doctrine of identity.

## 3 Dealing with Death and Finitude

A further question to ask regarding this doctrine of identity is how to make sense of this position in the larger context of Schrödinger's life. In this chapter, I argue that a closer study of Schrödinger's published and unpublished writings shows how this idea of Indian origin offered Schrödinger a form of consolation—a way of dealing with death and the finitude of existence.

In his book, *My View of the World*, Schrödinger explicitly mentioned that the doctrine of identity offered religious consolation.^70^ A look into Schrödinger's personal notebooks sheds light on what he may have meant.

**Figure 2:**
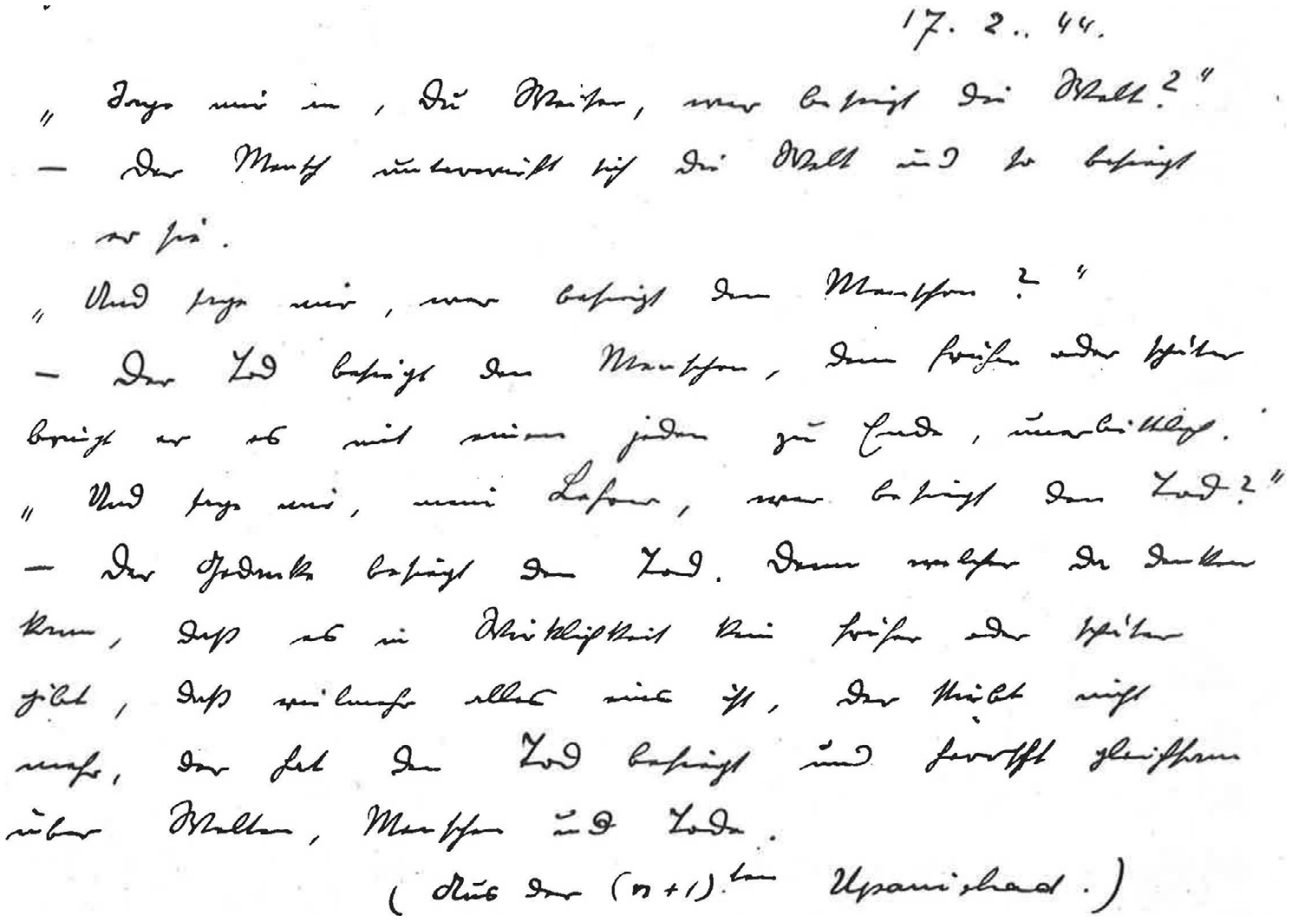
Excerpt from Schrödinger's diary dated 17 February 1944.^74^ This is the original German version of his self‐written Upaniṣad, before he translated it to English. Erwin Schrödinger, Diary 1942–1944 [Ephemerides], 1944, Austrian Central Library for Physics, Vienna/Erwin Schrödinger Archive, L33‐557, copy, in parts transcribed by Auguste Dick.

Schrödinger wrote many notebooks dedicated to physics and a decent amount dedicated to Indian thought. Moreover, he kept collections of notebooks throughout his life writing on his love life, (the politics of) war, and all types of day‐to‐day things. One of the themes that can be identified running through these diaries concerns his thoughts on death and the finitude of existence, especially around the time of the Second World War. In 1940, for example, Schrödinger wrote about how “Individuals have to escape death […] they have to fear it,”^71^ as fear of death is a prop that is introduced to secure the survival of the species.^72^ What is more, his musings on death and existence were often interlaced with references to the doctrine of identity. For example, on February 17, 1944, Schrödinger wrote his own Upaniṣad in his notebook (**Figure** **2**). After a couple of iterations in German, he added an English translation of his own Upaniṣad:

Tell me, enlightened one, who overpowers the world?

– Man subjects all this world to his command.

and thus he acquires power over it.

And tell me, who has power over man.

– Death has. For sooner or later he puts an end to it with every man, inexorably.

And tell me, my teacher, who conquers death?

– Thought conquers death. For he who is able to think, that in reality there is no sooner or later, that rather all that is one, he dies not, he has conquered death and, as it were, reigns over worlds, men and death.–

E.S.^73^

Schrödinger, here, captured aspects of the Upaniṣads in both thematic and style. Death, for Schrödinger, can be conquered by realizing (or knowing) the underlying unity of past, present, and future.^75^ This idea seems more than something he merely conceptually accepted, it seems to bear some personal meaning to him. When his dog—Burschi—died on May 8, 1946, for example, Schrödinger wrote that through friendship with the dog, we transcend our own sphere and bind ourselves to eternity.^76^ In reality, he elaborated, both the dog and humans are already eternal.^77^ It is again this underlying unity in which people, animals, and the world are united that Schrödinger referred to when dealing with the death of his dog.^78^ Schrödinger thus found in this Advaitin emphasis on ultimate unity an answer to the perceived finitude of existence. The doctrine of identity, Schrödinger seems to have maintained, provided insight into the liberation from death through the universal and eternal character of the self, and the unity of this universal self with the world.

## 4 The Doctrine of Identity in Schrödinger's Philosophical Views

**Figure 3:**
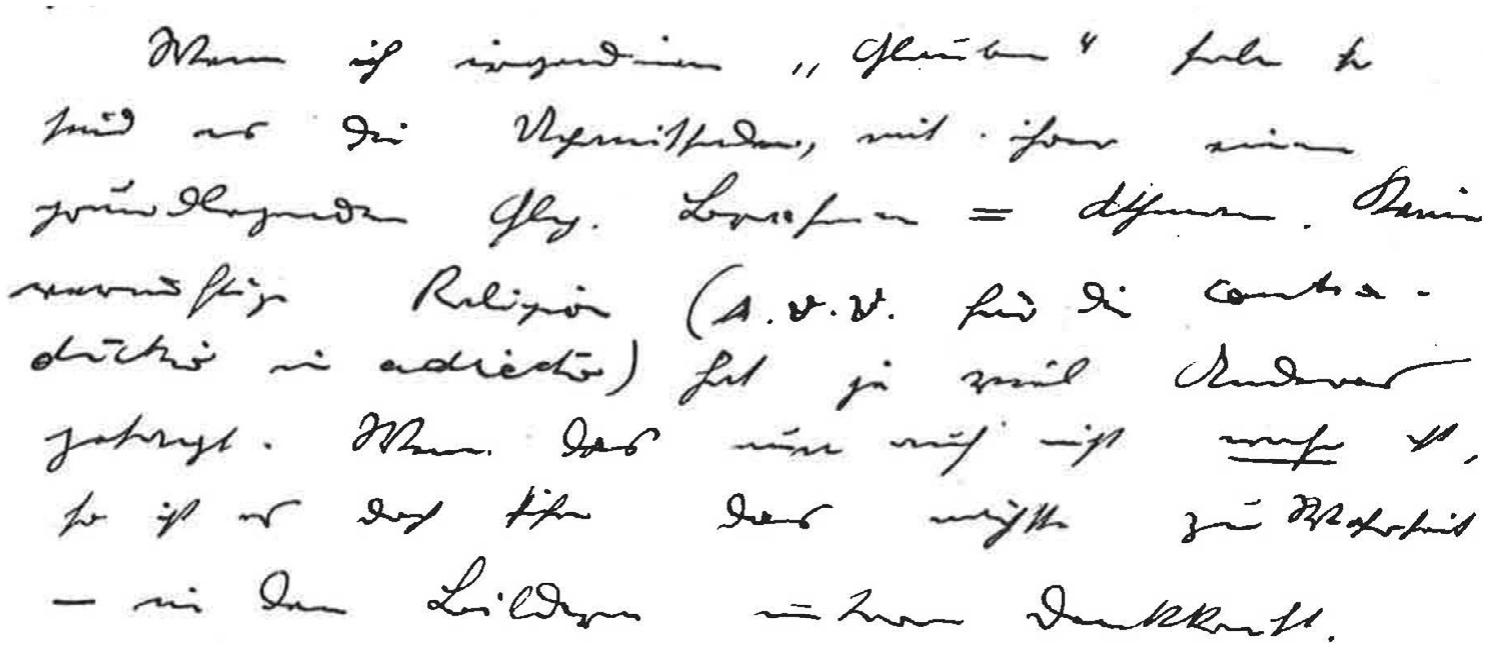
Excerpt from Schrödinger's diary dated June 1, 1941, where he wrote about his belief in the Upaniṣadic message.^85^ Erwin Schrödinger, Diary 1940–1941 [Ephemerides], 1941, Austrian Central Library for Physics, Vienna/Erwin Schrödinger Archive, L33‐557, copy, in parts transcribed by Auguste Dick.

In a diary entry dated the first of June 1941, Schrödinger wrote that if he had any belief, he believed in the Upaniṣadic message that *ātman* (universal self) equals *brahman* (ultimate totality of reality) (**Figure** **3**).^79^ Concerning this insight, he reasoned that even if this statement is also not true, it is “certainly the closest thing to the truth […].”^80^ One might then wonder whether Schrödinger expressed here his belief in the doctrine of identity as a religious belief or a philosophical conviction. The exact status of religion in Schrödinger's thought remains rather puzzling, as he often used religious symbolism in his philosophical texts but claimed to be an atheist, as other scholars have noted.^81^ In the remainder of this paper, I do not attempt to spell out the role of religious versus philosophical convictions in Schrödinger's thinking (and whether such a distinction should be maintained at all in Schrödinger's thought). However, I will argue in the remainder of this section that Schrödinger seemed to view this doctrine of identity as a key philosophical notion, adopting it in metaphysical reflections on the nature of reality.^82^ Schrödinger categorized this doctrine of identity explicitly as a “mystical” and “metaphysical” thesis^83^—in the same way as, according to him, a position such as materialism is a mystical and metaphysical thesis.^84^ I will address, for Schrödinger, the philosophical significance of two notions that are present in this doctrine of identity, particularly the notion of non‐dualism and a conventional‐ultimate distinction, and highlight instances where these notions find resemblances in his philosophy of science.

### 4.1 The Role of Non‐Dualism

**Figure 4:**
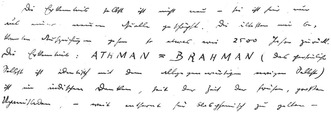
Excerpt from Schrödinger's diary dated March 10, 1944, where he mentioned how his point—about the self being identical to the world—is nothing new, as it is found in the Upaniṣadic slogan *ātman* = *brahman*.^93^ Erwin Schrödinger, Diary March−July 1944 [Ephemerides], 1944, Austrian Central Library for Physics, Vienna/Erwin Schrödinger Archive, L33‐557, copy, in parts transcribed by Auguste Dick.

In another noteworthy diary entry, dated March 10, 1944, Schrödinger suggested the metaphysical significance of this non‐dualist notion expressed in his doctrine of identity. Schrödinger wrote about how the “I” (the universal self) moves the atoms according to the laws of physics.^86^ His reasoning can be summarized as follows. Schrödinger, in this entry, starts with two observations concerning his body: 1) the body functions as a pure mechanism, obeying the laws of nature; and 2) Schrödinger notes that “I” (Schrödinger) am directing that body. About 1) he is reasonably certain; and about 2) he is absolutely certain through “irrefutable direct experience.”^87^ Putting these observations together, Schrödinger argued it follows that “I,” in the broadest metaphysical sense of the word (i.e., all conscious agents to have ever existed, Schrödinger noted), am the one who directs and regulates the motions of atoms.^88^ Schrödinger elaborated that this thought is not new and attributes it to the Upaniṣads (**Figure** **4**).^89^ Moreover, Schrödinger noted that although this is blasphemy in Christian circles, he concluded just like he did in his book *What Is Life?*,^90^ that not only the Upaniṣads but also “the biggest mystics of all time” arrived at “*deus factus sum* (I have become god—I have become personally identical with god).”^91^ We see Schrödinger here utilize religious terminology and symbolism in order to express metaphysical reflections about the nature of reality (on that which is beyond the movement of atoms and the laws of nature)—coupling his insights on the universal self to the movement of atoms. Furthermore, it seems Schrödinger imbued, here, this doctrine of identity with a universal character—the biggest mystics, regardless of time and culture, all arrived at versions of this underlying unity of self and world. This universality of these ideas was underlined in his book *Mind and Matter*, where Schrödinger wrote that his view “levels with that of Aldous Huxley” in his book *The Perennial Philosophy*.^92^

Schrödinger discussed the philosophical (and scientific) significance of this non‐dualist notion in the doctrine of identity not only in his personal notes. This theme is easily recognized in his published writings.^94^ In his book *Mind and Matter*, where Schrödinger once again discussed the doctrine of identity, he mentioned how this idea of unity of mind and matter is particularly unpalatable in Western thought and science.^95^ This is because, he reasoned, Western thought—based on Greek “objectivation”—has cut itself off from the mind, the subject.^96^ He called this the “exclusion principle,”^97^ elaborated as: “we exclude the Subject of Cognizance from the domain of nature that we endeavor to understand.”^98^ Schrödinger again then introduced his paradox (how to make sense of the plurality of minds) and stated: “There is obviously only one alternative, namely the unification of minds or consciousnesses. Their multiplicity is only apparent, in truth there is only one mind. This is the doctrine of the Upanishads.”^99^ Schrödinger then connected the doctrine of identity to his ruminations about the omission of the subject in Western thought and science. He wrote: “I do believe that this is precisely the point where our present way of thinking does need to be amended, perhaps by a bit of blood‐transfusion from Eastern thought.”^100^ Schrödinger suggested a blood transfusion of Eastern thought into the West aimed specifically at assimilating the doctrine of identity with Western thought: introducing the mind, or “I,” into Western thought and science.^101^ Schrödinger here makes a prescriptive argument: this doctrine of identity is not just an accurate philosophical thesis, Western thought and science *should* take note of this doctrine of identity. While in colonial India people such as late nineteenth and early‐twentieth century scientist Jagadish Chandra Bose attempted to assimilate traditions such as Advaita Vedānta with Western science, Schrödinger did not work out in much detail what such a “blood transfusion” would look like.^102^ However, his arguments display his commitment to take this doctrine of identity seriously in contemporary philosophical and scientific reflections and, furthermore, display a commitment to take some Advaita Vedānta insights seriously more broadly in philosophy and science.

It thus becomes clear from both published and unpublished writings that Schrödinger took this non‐dualist notion seriously in his metaphysical ruminations about the nature of reality. Moreover, I argue there is reason to believe this non‐dualist position situated some reflections in his philosophy of science, particularly his reflections on the subject–object relation in his philosophy of quantum mechanics. In *Mind and Matter*, Schrödinger replied to a claim of what he called the “prevailing school of thought in quantum physics” at the time, namely that of “Niels Bohr, Werner Heisenberg, Max Born and others.”^103^ In Schrödinger's construal the abovementioned authors argued that under the impact of our refined methods, quantum mechanics showed us how the boundary between subject and object collapsed—as the object is affected by our observation.^104^ Schrödinger responds elaborately, arguing that ultimately “subject and object are only one. The barrier between them cannot be said to have broken down as a result of recent experience in the physical sciences, for this barrier does not exist.”^105^ In the chapter immediately following this reply to Bohr, Heisenberg, and Born, Schrödinger offered his remedy against excluding the subject, or the mind, from nature, namely “Eastern thought.”^106^ We see, here, how it is precisely this tenet of the unity of subject and object—this position of ultimate non‐duality of subject and object he formulated time and time again by Schrödinger through the Advaita insight of *ātman* equals *brahman—*which seems to motivate this instance of Schrödinger's response to Bohr, Heisenberg, and Born.

Interestingly, since the postulation by Bohr, Heisenberg, and Born (that Schrödinger rejected) casts doubt on the sharp distinction between subject and object in quantum physics, Schrödinger could have used that postulation precisely as a demonstration of the point he tried to convey, namely that quantum mechanics shows the subject should be included in our thinking. But Schrödinger disagreed with their reasoning more fundamentally. Saying this subject–object relation breaks down in quantum mechanics suggests the original dualist assumption was justified in other settings. Schrödinger critiqued this by employing a more thorough and encompassing reading of the non‐dualist thesis. Only for practical reference, to navigate everyday life, do we act as if there is a distinction between subject and object, Schrödinger proclaims.^107^ But we should not say the subject–object relation breaks down at the quantum level, as, for Schrödinger, this relation does not make sense at any ultimate level. There is ultimately only non‐duality. Schrödinger's insistence on non‐duality thus situated his reflection on the subject–object relation in quantum mechanics. In this instance, it framed his reply to other physicists at the time.

### 4.2 The Perceived and the Ultimate

Besides this emphasis on non‐dualism, Schrödinger also seems to have utilized a distinction that resembles one in Śaṅkara's Advaita Vedāntin works: that between conventional, perceived reality (*vyāvahārikasattā)* and ultimate, non‐dual reality (*pāramārthikasattā)*. For Śaṅkara, conventional reality is the world of everyday, empirical reality, in which we perceive a plurality of objects and people.^108^ This reality, for Śaṅkara, is illusory, but that is not to say it is totally unreal or non‐existent. Rather, it exists *as a convention* but is illusory in an ultimate sense.^109^ The word *illusion*, thus, refers to the sense in which there is no ultimate metaphysical character to the entities we observe. A frequently cited analogy is Śaṅkara's idea that one can mistake a rope for a snake: the observation of the snake is real in some sense, as it has real effects on us (e.g., can induce a physical reaction), but ultimately there is no snake, so the observation of the snake is illusory in an ultimate sense.^110^ In the analogy, the observation of the snake is conventionally real but has no ultimate character, as there is no snake. Similarly, the world we observe around us, with its plurality of individual minds and objects, exists conventionally for Śaṅkara but has no ultimate metaphysical character. In the following passages, as well as in various other instances across his published and unpublished writings,^111^ Schrödinger seems to adopt a similar distinction. The distinction between perceived (but deceptive) plurality and the ultimate reality of monist unity is key to what Schrödinger expressed as “the profound rightness of the basic conviction in Vedanta” in 1925, namely, that “the plurality that we perceive is only an appearance; it is not real” followed by the conclusion that you are “all in all.”^112^ And in 1960, in the second part of *My View of the World*:

[…] astronomy gives us of myriads of suns with, perhaps, inhabitable planets, and of a multitude of galaxies, each with myriads of such suns, and ultimately of a probably finite universe […], mediated to our senses by the indescribable panorama of the starry heavens on a clear night. To me personally all that is maya, albeit maya of a very interesting form, exhibiting laws of great regularity.^113^

Central to this comment is the deceptive quality of *māyā* that Schopenhauer, Deussen and Schrödinger took from Śaṅkara's definition of *māyā*, as discussed in Section 2. Schrödinger hinted more directly at what this means in his 1944 book *What is Life?*:

The only possible alternative is simply to keep to the immediate experience that consciousness is a singular of which the plural is unknown; that there is only one thing and that what seems to be a plurality is merely a series of different aspects of this one thing, produced by a deception (the Indian MAJA).^114^

Schrödinger here seems to have utilized a similar distinction Śaṅkara maintained. For Schrödinger, we perceive a plurality of things and beings, but this is deceptive or illusory in an ultimate sense, as there is ultimately only the singular consciousness. Note, here, that the distinction between the perceived and the ultimate is, for Schrödinger, not a metaphysical distinction in the sense that these are two metaphysically distinct worlds (see also the discussion on scientific realism later in this section). Schrödinger explicitly used the English word “illusion” in some instances.^115^ Note, furthermore, that Schrödinger's two definitions of the self discussed in Section 2.3 follow a similar perceived‐ultimate structure. Moreover, also his views on the subject–object relation discussed in Section 4.1 can be understood through this perceived‐ultimate distinction: the subject–object distinction can be expressed precisely as a conventional distinction in Śaṅkara's terms, one that can be used for reference in daily life, but should be rejected in an ultimate account of reality—as subject and object are ultimately one.

The perceived‐ultimate distinction,^116^ I would argue, helps clarify certain ways in which Schrödinger's philosophical views can be construed, particularly the relation between his reading of Mach's neutral monism and his embrace of Advaita monism. Some contemporary historians of Mach stress that Mach's neutral monism should not be read as a form of atomism, as these neutral elements for Mach are not atomistic logically independent simples but are rather “always bound up in functions that supervene on their causal powers to affect each other.”^117^ Insofar as I can tell, Schrödinger read Mach's neutral elements not as atomistic in the material sense (as he stresses these neutral elements can constitute either mind or matter). However, there seems to be a sense in which Schrödinger construes these elements as independent simples. Schrödinger referred to these elements as “primitive elements” constituting “the external world and consciousness.”^118^ Moreover, in his book *Nature and the Greeks*, Schrödinger describes how the “real world around us” and “we ourselves” are constituted “of the same bricks” and argues that we can think of these neutral elements either as constituting mind or the material world, but not both—similar to how a child can build various things with a box of bricks, such as a house or a tower, but it cannot build both a house and a tower with these objects at the same time.^119^ In Chapter VII of *My View of the World*, Schrödinger argues that such notions of Mach (and Avenarius and Schuppe) come “as near to the orthodox dogma of the Upanishads as it could possibly do without stating it *expressis verbis*.”^120^ A potential problem in Schrödinger's thinking now presents itself: how can Schrödinger maintain there is ultimately only a unitary *brahman* (which for Śaṅkara means there is no way one can ultimately make sense of plurality, which Schrödinger explicitly adopts with his emphasis on non‐plurality in his “Vedantic Vision”)^121^ while also maintaining there is a collection (i.e. plurality) of primitive elements? I argue that looking at Śaṅkara's conventional‐ultimate distinction, which Schrödinger seemed to adopt in his distinction between the perceived and the ultimate, allows for a reading that reconciles these two ideas in Schrödinger's philosophy.

Schrödinger construing the “real world around us” and “we ourselves” through primitive elements can be understood as him using neutral monism to conceptualize conventional, perceived reality. When Schrödinger introduced this notion of the “real world around us,” he did so in a rather ironic fashion—as he was critiquing, in the chapter, our tendency to understand the world around us as if it were something distinct from ourselves, which is mistaken according to Schrödinger.^122^ Consider again Schrödinger's claim in the aforementioned passage: “[…] what seems to be a plurality is merely a series of different aspects of this one thing.”^123^ In this assertion, the neutral elements can now be understood as the *aspects* (i.e., the perceived distinct phenomena, conventional reality, the “real world around us”), while that which they are aspects of, “this one thing,” is the ultimate monist unity (*brahman*, *ātman*, universal consciousness or the universal self). This aligns with Schrödinger's characterization of neutral elements as “sense perceptions, memory images, imagination, thought,”^124^—these typifications concern conventional, perceived reality, which is ultimately happening in the unitary consciousness.^125^ In this way, if we grant Schrödinger the principle of charity and expect he would have noticed that one cannot maintain there is ultimately one thing while there are also a plurality of elements, we can understand Schrödinger's philosophical views as accepting his reading of Mach's neutral monism to make sense of perceived reality (where the elements are the aspects), while the radical unity expressed in Śaṅkara's views is accepted by Schrödinger to describe reality in an ultimate sense (which is that which the elements are aspects of).^126^

This notion of a distinction between the perceived and the ultimate in the doctrine of identity is thus adopted in Schrödinger's philosophical reflections on the nature of reality and helps make sense of his reading of Mach's neutral monism in his thinking. Important to note is that even if one would disagree with my analysis of the relation between Schrödinger's Advaita beliefs and his use of neutral monism, the very fact that Schrödinger discussed this doctrine of identity in relation to the philosophical views of people such as Mach (one of his major influences in physics and philosophy) further underlines the philosophical significance of this doctrine of identity for Schrödinger.

Lastly, the perceived‐ultimate distinction that Schrödinger often expressed in Indian terminology resonates further with some of Schrödinger's reflections in the philosophy of science, particularly his attitude to scientific realism. In his book, *Schrödinger's Philosophy of Quantum Mechanics*,^127^ Michel Bitbol uses Blackburn's notion of quasi‐realism^128^ to capture the more mature scientific realist position Schrödinger arrived at around the 1950s, developed over the years. A quasi‐realist is “someone who, starting from an anti‐realist position finds himself progressively able to mimic the thoughts and practices supposedly definitive of realism.”^129^ In essence, Schrödinger's more sophisticated scientific realist position was metaphysically strictly and unambiguously anti‐realist about the “real world around me” (or as Bitbol puts it: “the reality of the physicist”)^130^ but was methodologically realist.^131^ In 1954, Schrödinger talked about the “real world around me” as something he constructs.^132^ In Bitbol's book, one conclusion posed is that when Schrödinger talks about scientific entities as existing in a real sense, he talks about them as existing *as constructs*.^133^ Schrödinger thus maintained a rather loose definition of existence—one where a conceptual entity posited by the scientist exists already *as* this conceptual entity. This allowed Schrödinger to treat scientific entities as objects of study with utter seriousness while not necessarily ascribing a metaphysical character to them. Bitbol emphasized that this scientific anti(‐metaphysical) realist view that Schrödinger holds regarding the “real world around us” includes the way Schrödinger understands conceptual entities in physics.^134^

Bitbol mentioned Indian thought briefly when discussing Schrödinger's scientific realist position,^135^ but a resonance with Śaṅkara's philosophy can now be made more explicit. In Schrödinger's scientific realist considerations, the emphasis on having a world picture that Bitbol points out clearly has Viennese roots, particularly with Boltzmann.^136^ However, I want to highlight that this idea—that scientific entities can be thought of as real in the sense that they are real as constructed entities—resonates with the way Śaṅkara construes distinct entities as existing by convention. Both Śaṅkara's emphasis on conventional versus ultimate existence and Schrödinger's later quasi‐realist position emphasize the reality of (scientific) entities as conceptual constructs, as constructs we can (and should) talk and think about for pragmatic purposes, while they both fanatically deny the ultimate metaphysical character of these constructs in light of ultimate non‐duality.^137^ Note, lastly, that there is a sense in which both Śaṅkara and Schrödinger emphasize that appearances are constructed and that it is a lack of knowledge, or ignorance, that is involved in the construction of these appearances.^138^

Despite the influence of Kant on Schrödinger (and his generation of scientists), and a possible resonance between Schrödinger's abovementioned views and Kantian insights (e.g., think of the construction of the phenomenal world),^139^ a thorough study on the ways Kantian thought converges with Indian thought in Schrödinger is beyond the scope of this paper.^140^ In this paper, I merely argue that the resemblance between Śaṅkara and Schrödinger should not be left unnoted. It is especially relevant to mention the resemblance with Śaṅkara since Schrödinger chose to express his thoughts on the reality of the world and objects in it often in Advaita Vedānta terms and concepts (such as *māyā*, *brahman*, and *ātman*), in both his published and unpublished documents.

## 5 Conclusion

In this paper, I have historically contextualized Schrödinger's reading of Indian philosophy and shown that Schrödinger's doctrine of identity essentially captures Śaṅkara's Advaita Vedānta reading of the Upaniṣads. Furthermore, I have indicated that the insights of ultimate unity seem to have offered Schrödinger a form of religious consolation—a way of dealing with death and the finitude of existence. However, I have further shown how these insights offered Schrödinger more than religious comfort; he takes the doctrine of identity seriously in his philosophical views. Schrödinger adopts the doctrine of identity into his metaphysical ruminations about the nature of reality, and it further resonates with some of his reflections in the philosophy of science, such as the subject–object relation (in quantum mechanics) and his views on scientific realism.

Although the generality of the grand insights adopted from India might obscure a possible influence on Schrödinger's physics, the intensity with which Schrödinger embraced these ideas does suggest the relevance of further research on this topic.^141^ Furthermore, Schrödinger preached that metaphysics should be the basis for science, so it would be interesting to develop a better understanding of whether and to what extent Schrödinger's own metaphysical views served as the basis for his physics. The fanatic embrace of Indian ideas by Schrödinger shown in this paper demonstrates that Indian insights should not be excluded from such an assessment of philosophical ideas in Schrödinger's scientific pursuits.

## Footnotes

1
Schrödinger wrote: “In 1918, when I was thirty‐one, I had good reason to expect a chair of theoretical physics at Czernowitz (in succession to Geitler). I was prepared to do a good job lecturing on theoretical physics, […] but for the rest, to devote myself to philosophy, being deeply imbued at the time with the writings of Spinoza, Schopenhauer, Mach, Richard Semon and Richard Avenarius. My guardian angel intervened: Czernowitz soon no longer belonged to Austria. So nothing came of it. I had to stick to theoretical physics, and, to my astonishment, something occasionally emerged from it. So this little book is really the fulfilment of a very long cherished wish.” Schrödinger 1964, on p. viii.

2
This is the English translation of the book *Meine Weltansicht* originally published in 1961. I have access to the German text in its later publication in *Mein Leben, meine Weltansicht*, see Schrödinger 1985. The book consists of two parts: the first part is written in 1925, and the second part written in 1960. The original text on which Schrödinger based the chapter “The Vedantic Vision” in the first part can be found in a notebook dated 1925. For the original notebook, see Erwin Schrödinger, Diary 1925, 1925, Austrian Central Library for Physics, Vienna/Erwin Schrödinger Archive, L33‐705/8.

3 Schrödinger 1964, on p. 18. This “paradox” pops up in several of Schrödinger's writings. In a magazine article, in Schrödinger 1961, on p. 47, he calls this “Die eigentliche Schwierigkeit für die Philosophie” (“the real difficulty for philosophy”). The translations in this paper from Schrödinger's published or unpublished sources are my own unless otherwise specified.

4 Schrödinger 1964, on p. 18.

5 Ibid., on pp. 21–22.

6 Ibid., on p. 21.

7 Ibid., on p. 92. Similar insights appeared all over his philosophical writings and personal notebooks. Moreover, according to Bitbol 1998, on p. 13, Schrödinger called the Upaniṣadic slogan “Atman equals Brahman” half‐smilingly “the second Schrödinger equation” and supposedly ended his conference talks with this same Indian slogan.

8 Schrödinger 1964, on pp. 3–7.

9 Schrödinger 1964; Schrödinger 1992; Schrödinger 1996.

10 Schrödinger 1992, on p. 130.

11 Wessels 1975, on pp. 73–109. Wessels concisely mentioned Schrödinger's interest in Indian thought.

12 Moore 1989, e.g., on p. 172.

13 Moore 1989, on p. 348.

14 Bitbol gave a talk at the French embassy in India in 1998, hereafter referenced to as Bitbol 1998. I have accessed the transcript of the talk via Bitbol's online publication on Acadmia.edu, https://www.academia.edu/29024231/SCHRÖDINGER_AND_INDIAN_PHILOSOPHY?rhid=27605384732&swp=rr‐rw‐wc‐45565806 (accessed 10 April 2024). Unless otherwise indicated, the page numbers I refer to when I refer to Bitbol's 1998 talk are the page numbers present in the transcript document.

15 Here quoted after an “amplified version” of the 1998 talk mentioned in note 16, published online (undated) as a separate document. Michel Bitbol at Academia.edu, on 25–26, https://www.academia.edu/45565806/Erwin_Schrödinger_an_approach_of_mind_and_matter_inspired_from_the_Upanishads_and_from_Buddhism&nav_from=0b05daa6‐75a0‐436c‐9789‐e4a98ff2dbdc&rw_pos=0 (accessed 10 April, 2024).

16 Bitbol 1998, on p. 20. While I depart from Bitbol's diagnosis in specific matters (particularly his conclusions on the role of Buddhism in Schrödinger's conception of the personal self, addressed in Section 2.3), I essentially underwrite Bitbol's judgment regarding the importance of Advaita Vedānta in Schrödinger's thought.

17 While Moore did differentiate between different readings within Vedānta, he wrote that all forms of Vedānta agree that “things and selves do not have individual existences, but are all joined together as effects of Brahman, or in brief are Brahman itself”—he referred to this message of unity throughout his book interchangeably as a Vedāntic or Upaniṣads message, while, as we will see later in this paper, this emphasis on unity is specifically made by Advaita Vedāntin thinkers. Moore 1989, e.g., on pp. 171–173.

18 Bitbol referred to both Vedānta and Advaita Vedānta throughout Bitbol 1996, Bitbol 1998.

19 More specifically, Schrödinger's reaction to Niels Bohr, Werner Heisenberg, and Max Born on the subject‐object relation in his book *Mind and Matter*, in Schrödinger 1992 (first published in 1958).

20 Schrödinger admired the work of, among others, Epicurus, Baruch Spinoza, Arthur Schopenhauer, Richard Semon, Immanuel Kant, Ernst Mach, Ludwig Eduard Boltzmann, and Patrick Lafcadio Hearn.

21 Erwin Schrödinger, Diary 2 July 1918, 1918, Austrian Central Library for Physics, Vienna/Erwin Schrödinger Archive, L33‐705/3.

22 Schrödinger wrote: “[…] ich hatte soeben mit großer Begeisterung Schopenhauer und durch ihn die Einheitslehre der Upanishaden kennengelernt.” Schrödinger 1985 on p. 18.

23 Moore 1989, on pp. 111 and 172. Moore did not provide a source for why he wrote Schrödinger read every word of Schopenhauer. We could read Moore's statement about Schrödinger reading every word of Schopenhauer as an attempt to express his perceived extent of Schrödinger's engagement with Schopenhauer's thought, rather than a factive statement meant to be read literally.

24 Ibid., on p. 112.

25 Anquetil‐Duperron 1801.

26 Merkel 1948, on p. 164.

27 Halbfass 1988, on p. 33. See also ibid., on pp. 32–35.

28 Within the Indian philosophical tradition of Vedānta, the combination of the Upaniṣads, the *Brahma‐sūtras* and the *Bhagavadgītā* together form the philosophical foundation of systematic Vedānta (these works form the *prasthānas* or *prasthāna‐traya* as the triple canon of Vedānta).

29 Note here, that although the reading that non‐dualism should be understood as monism is widely shared in academic circles, it is not universally accepted, e.g., see Berger 2021. For the purpose of this paper, I follow the widely accepted reading of Śaṅkara.

30 Halbfass 1988, on p. 34

31 E.g., in Nicholls 1999, Copleston 1987, on p. 216.

32 Anquetil‐Duperron read the Upaniṣads as an idealist philosophy maintaining everything outside of the self is merely phenomenological appearance, see Berger 2000, on p. 70—resulting in a form of illusionism resembling the form of illusionism found in Advaita Vedānta. Anquetil‐Duperron, furthermore, believed this philosophy of the Upaniṣads to be compatible with Kantian idealism, which, according to ibid., on xiv–xv and 70, formed a “guiding principle in Schopenhauer's reading.”

33 Ibid., e.g., on pp. iv–v.

34 Ibid., e.g., on p. 37.

35 Ibid., e.g., on p. 130.

36 While, in the Upaniṣadic texts, *māyā* is often understood as the power to create the world, see ibid., on p. 71.

37 This criticism concerns more than his use of *māyā* and his privileging of Advaita readings in his use of the Upaniṣads. Schopenhauer is critiqued for equivocating and generalizing various Indian concepts, such as *brahman* and *tat tvam asi*, and comparing these in an unsophisticated way to similarly generalized notions in Western thought. See ibid., e.g., on pp. 7 or 233.

38 Ibid., e.g., on pp. xvi–xviii.

39 Schrödinger 1964, on pp. 109–110.

40 Moore noted Schrödinger wrote this around 1918 but did not elaborate on why he believed that. Moore 1989, on p. 113. However, there is reason to believe that this notebook where Schrödinger listed these sources should indeed be dated around 1918, as Schrödinger here lists only one of Paul Deussen's books, published in 1906. However, we know for a fact that Schrödinger later read other works of Deussen, published in 1919 and 1921, as Schrödinger refers to these later published sources in his 1960 book *Meine Weltansicht*. See Schrödinger 1964, on p. 96.

41 Berger 2000, p. 310. Erwin Schrödinger, [Indische Philosophie und Religion], undated, Austrian Central Library for Physics, Vienna/Erwin Schrödinger Archive, L33‐705/2.

42 Erwin Schrödinger, [Indische Philosophie und Religion], undated, Austrian Central Library for Physics, Vienna/Erwin Schrödinger Archive, L33‐705/2.

43
Deussen 1906. It is not clear from the diary what traditions Schrödinger categorizes under “Brahmanism.” However, given that only Deussen's work on Vedānta is mentioned in this list, it seems, at least in this stage of his life, that Schrödinger only refers to Vedānta here. The German words Schrödinger uses for these lists are “Allgemein,” “Buddhismus,” and “Brahmanismus.” Erwin Schrödinger, [Indische Philosophie und Religion], undated, Austrian Central Library for Physics, Vienna/Erwin Schrödinger Archive, L33‐705/2.

44 Deussen 1906. This presence of Śaṅkara in Deussen's work has also been noted by other scholars. Berger 2000, on pp. xviii and 5.

45 Schrödinger 1964, on p. 18.

46 Ibid. For the “real difficulty” citation, see Schrödinger 1961, on p. 47.

47 Schrödinger 1964, on p. 37.

48 Ibid., on pp. 96 and 98–99. To my knowledge, there is no record of when exactly Schrödinger got access to these sources of Deussen. The works of Deussen referred to here are published in 1919 and 1921, indicating Schrödinger probably got access to these books somewhere between 1919 and 1960.

51 Halbfass 1988, on p. 35.

50 Note, here, that although Schrödinger could have known about some of the problems with Schopenhauer's and Deussen's work through reading scholars such as Max Müller, it is only since Wilhelm Halbfass 1988 (long after Schrödinger died) and later emulated by scholars such as Douglas Berger that the deep‐rooted role of Śaṅkara's work has become more visible. So, although Schrödinger could have known about some of the misconceptions in Schopenhauer and Deussen, it is possible he was not aware of the intricacies.

51 Schrödinger 1964, on p. 101.

52 In his book *What is Life?* published in 1944, see Schrödinger 1992, on p. 89; in his book *Mind and Matter* published in 1958, see ibid., on p. 129; and again in the parts of *Meine Weltansicht* that were written in 1960, see Schrödinger 1964, on pp. 99 and 101.

53 Schrödinger 1964, on p. 101.

54 For the sake of clarity, I add two brief comments on the notion of continuity I address here. As there are various debates and various differing opinions on the diagnosis of continuity in Schrödinger's thought in general, I emphasize that I am here merely observing a continuity when it comes to two specific notions in Schrödinger's use of Indian philosophy, namely, in Schrödinger's use of the notion of *māyā* (which appears to be the Advaita Vedānta notion), and in the central idea Schrödinger aimed to convey in what he called the doctrine of identity (similarly inspired by the Advaita emphasis on ultimate unity).

55 Note that Schopenhauer should have been aware that Anquetil‐Duperron's translation was highly criticized, but he chose to follow it anyway. Berger 2000, on p. 69.

56 Bitbol 1998.

57 Wessels 1975, on p. 84.

58 Moore 1989, on p. 171.

59 Ibid., on pp. 171–172.

60 E.g., Schrödinger 1964, on p. 13. Also, see: Erwin Schrödinger, [Indische Philosophie und Religion], undated, Austrian Central Library for Physics, Vienna/Erwin Schrödinger Archive, L33‐705/2.

61 Schrödinger wrote that Lafcadio Hearn, a writer in Zen Buddhism, exerted an influence on him, in Erwin Schrödinger, Diary 2 July 1918, 1918, Austrian Central Library for Physics, Vienna/Erwin Schrödinger Archive, L33‐705/3.

62 Schrödinger writes: “das ich […] ist sicher vergänglich.” Erwin Schrödinger, Diary March−July 1944 [Ephemerides], 1944, Austrian Central Library for Physics, Vienna/Erwin Schrödinger Archive, L33‐557, copy, in parts transcribed by Auguste Dick.

63 “augenblicklich richtige, […] aber mehr zufällige.” Ibid.

64 “Auf der anderen Seite ist Ich aber auch gleich mit Es.” and “Ich bin Es. Ich bin das unpersönliche Personalpronomen der dritten Person. Das Heißt Alles in Allem. − Und das ist wahr.” Ibid.

65 Śaṅkara in *Brahmasūtrabhāṣya* II, 3, 50, quoted after Date 1954, vol. 2, on p. 59.

66 E.g., Deutsch 1969, on p. 51.

67 E.g., Erwin Schrödinger, Diary 1940–1941 [Ephemerides], 1941, Austrian Central Library for Physics, Vienna/Erwin Schrödinger Archive, L33‐557, copy, in parts transcribed by Auguste Dick.

68 Transcription of Figure 1: “Ich bin Es. Ich bin das unpersönliche Personalpronomen der dritten Person. Das Heißt Alles in Allem. – Und das ist wahr.”

69 Bitbol 1998, on p. 7. Furthermore, Bitbol pointed out that Schrödinger talked about the personal self in a way that is similar to a Buddhist definition of the self through aggregates, see ibid.

70 Schrödinger 1964, on p. 107.

71 “Die Individuen müssen den Tod flüchten—und da sie nun einmal bewußt handeln, müssen sie ihn fürchten.” Erwin Schrödinger, Diary 1940–1941 [Ephemerides], 1940, Austrian Central Library for Physics, Vienna/Erwin Schrödinger Archive, L33‐557, copy, in parts transcribed by Auguste Dick.

72 “Besonders wenn man weiß, wie die Todesfurcht ein lediglich zwecks Arterhaltung eingeführtes Requisit ist.” Ibid.

73 Erwin Schrödinger, Diary 1942–1944 [Ephemerides], 1944, Austrian Central Library for Physics, Vienna/Erwin Schrödinger Archive, L33‐557, copy, in parts transcribed by Auguste Dick.
74 Transcription of Figure 2: “17.2.44. ‘Sage mir an, Du Weiser, wer besiegt die Welt?’ – Der Mensch unterwirft sich die Welt und so besiegt er sie. ‘Und sage mir, wer besiegt den Menschen?’ – Der Tod besiegt den Menschen, denn früher oder später bringt er es mit einem jeden zu Ende, unerbittlich. ‘Und sage mir, mein Lehrer, wer besiegt den Tod?’ – Der Gedanke besiegt den Tod. Denn welcher da denken kann, daß es in Wirklichkeit kein früher oder später gibt, daß vielmehr alles eins ist, der stirbt nicht mehr, der hat den Tod besiegt und herrscht gleichsam über Welten, Menschen und Tode. (Aus der (n + 1)ten Upanishad.)”

75 Schrödinger, here, emphasized one‐ness through a temporal dimension. He argued one can conquer death through a presentist realization that what we understand as past and future is encapsulated in the now. Schrödinger's reasoning, here, resonated with how *brahman* is understood as temporal unity in, for example, the opening verses of the Māṅḍūkya Upaniṣad, in Olivelle 1996, on p. 289. Furthermore, Schrödinger's reasoning resembles how one attains immortality through knowledge as discussed in verse 10 and 11 of the Īśā Upaniṣad, in ibid., on 250.

76 “Durch die Freundschaft mit einem Tier überschreiten wir die eigenen Sphäre. […] Freundschaft mit dem Tier knüpft uns an die Ewigkeit. Denn in ihm erkennen wir sie. Das Tier ist ewig. (Wie wir selbst. Aber weil wir die Nase zu nah drauf haben und weil zu viel dummes Gewäsch darüber in Umlauf ist, erkennen wir es nicht.)” Erwin Schrödinger, Diary July 1945–October 1946 [Ephemerides], 1946, Austrian Central Library for Physics, Vienna/Erwin Schrödinger Archive, L33‐557, copy, in parts transcribed by Christof Capellaro and Leo Latten.

77 Ibid.

78 Schrödinger's dog was very dear to him, he wrote: “*Es leben vielleicht 3 oder 4 Menschen, deren Tod mir ebenso nahe ginge.*” Ibid.

79
Schrödinger wrote: “Wenn ich irgend einen ‘glauben’ habe, so sind es die Upanischaden, mit ihrer grundlegenden geg. Brahman = Atman. Keine vernünftige Religion […] hat je viel Anders gesagt.” Erwin Schrödinger, Diary 1940–1941 [Ephemerides], 1941, Austrian Central Library for Physics, Vienna/Erwin Schrödinger Archive, L33‐557, copy, in parts transcribed by Auguste Dick.

80 “Wenn das nun auch nicht wahr ist, so ist es doch sicher das nächste zur Wahrheit—in den Bildern unserer Denkkraft.” Ibid.

81 Moore 1989, on pp. 4, 112, and 409.

82 There are also implications for his philosophical views on ethics, which I will not address here. See, e.g., Schrödinger 1964.

83 Ibid., e.g., on p. 101.

84 Ibid., on pp. 93–94.

85 Transcription of Figure 3: “Wenn ich irgend einen ‘glauben’ habe, so sind es die Upanischaden, mit ihrer grundlegenden geg. Brahman = Atman. Keine vernünftige Religion (s.v.v. für die contradictio in adiecto) hat je viel Anders gesagt. Wenn das nun auch nicht wahr ist, so ist es doch sicher das nächste zur Wahrheit—in den Bildern unserer Denkkraft.”

86 “A) Mein Körper funktioniert als reiner Mechanismus, nach den Naturgesetzen. B) zugleich weiß ich aus unwiderleglicher unmittelbarer Erfahrung, daß ich seine Bewegungen dirigiere […]. Wenn man diese […] Tatsachen zusammenhält, so folgt bündig daß ich—ich im weitesten metaphysischen Sinn des Wortes, d.h. alle bewußt Handelnden die jemals ‘ich’ gesagt oder gefühlt haben—es folgt, sage ich, daß ich diejenige Person bin welche die ‘Bewegungen der Atome’ bestimmt und regelt, und zwar den allgemeinen Naturgesetzen entsprechend. (Was subjektiv mein Wille ist, ist Objektiv das Naturgesetz—so könnte man vielleicht—in exoterischer Sprechweise—sagen.)” Erwin Schrödinger, Diary March−July 1944 [Ephemerides], 1944, Austrian Central Library for Physics, Vienna/Erwin Schrödinger Archive, L33‐557, copy, in parts transcribed by Auguste Dick.

87 See ibid. for the transcript of the German text.

88 See ibid. for the transcript of the German text.

89 “Die Erkenntnis selbst ist nicht neu […]. Die ältesten mir bekannten Aufzeichnungen gehen so […] 2500 Jahre zurück. Die Erkenntnis: ATHMAN = BRAHMAN […] ist im indischen Denken seit der Zeit der frühen, großen Upanishaden […].” Ibid.

90 Schrödinger 1992, on p. 87.

91 “auch die großen Mystiker aller Jahr […] haben ihr großes Erlebnis, […] oftmals in Worten ausgedrückt, die sich auf den gemeinsamen Nenner bringen lassen: Deus factus sum (Ich bin Gott geworden—ich bin mit Gott persönlich identisch geworden.)” Erwin Schrödinger, Diary March−July 1944 [Ephemerides], 1944, Austrian Central Library for Physics, Vienna/Erwin Schrödinger Archive, L33‐557, copy, in parts transcribed by Auguste Dick.

92 Schrödinger 1992, on p. 90.

93 Transcription of Figure 4: “Die Erkenntnis selbst ist nicht neu, sie ist hier nur aus einer neuen Quelle geschöpft. Die ältesten mir bekannten Aufzeichnungen gehen so etwas wie 2500 Jahre zurück. Die Erkenntnis: ATHMAN = BRAHMAN (das persönliche Selbst ist identisch mit dem allgegenwärtigen Selbst) ist im indischen Denken seit der Zeit der frühen, großen Upanishaden—weit entfernt für blasphemisch zu gelten—.”

94 Most notably in his earlier referenced book *My View of the World*, Schrödinger 1964, Schrödinger presents this “Vedantic Vision” as a metaphysical thesis.

95 Schrödinger 1992, on pp. 128–130.

96 Ibid., on p. 130.

97 Ibid., on p. 122.

98 Ibid., on p. 118.

99 Ibid., on p. 129. Interestingly, Schrödinger here mentioned that forms of this doctrine of identity are not only found in the Upaniṣads, but also (for example) in Islamic Persian mystic traditions, e.g., Aziz Nasafi—aligning again with the universal character of this idea.

100 Ibid., on p. 130. Schrödinger added that such blood transfusion would not be easy, since precaution was needed to not to lose the logical precision reached in the West.

101 Notice the stark antithetical character of this claim to the dominating form of “mechanical objectivity” of late nineteenth century science, as described by Daston and Galison 2007.

102 I have not found historical records of Schrödinger interacting with or even knowing of Indian scientists such as Jagadish Chandra Bose. For more on Jagadish Chandra Bose and the cultural milieu at the time, see Brown 2016.

103 Schrödinger 1992, on p. 125. This point is also mentioned by Bitbol 1998. As it informs our notion of the role of non‐dualism in Schrödinger's thought, I should briefly mention it here.

104 Schrödinger 1992, on pp. 125–126.

105 Ibid., on p. 127.

106 Ibid., on pp. 128–130.

107 Ibid., on p. 127.

108 Hamilton 2001, on p. 127.

109 Note that the conventional‐ultimate distinction itself is not an ultimate distinction for Śaṅkara, as this would re‐introduce a certain duality: ultimately, there is only *brahman*. Note, furthermore, that it remains contentious in Śaṅkara scholarship as to how a supposedly changeless *ātman* could objectify itself into finite persons, see Mohanty 1993.

110 Śaṅkara discussed this example in the introduction of his *Brahmasūtrabhāṣya*, e.g., see Śaṅkara in Date 1954.

111
E.g., Schrödinger 1964; Schrödinger 1992; Erwin Schrödinger, Diary March‐July 1944 [Ephemerides], 1944, Austrian Central Library for Physics, Vienna/Erwin Schrödinger Archive, L33‐557, copy, in parts transcribed by Auguste Dick.

112 Schrödinger 1964, on pp. 16–21.

113 Ibid., on p. 107.

114 Schrödinger 1992, on p. 89.

115
For example, Schrödinger explicitly used the English word “illusion” when talking about the deceptive nature of the plurality we perceive in his book *Nature and the Greeks*, in Schrödinger 1996, on p. 29, where he writes: “Confronted with the intensely perceived oneness and changelessness of the Mind, the apparent kaleidoscopic character of the World had to give way and be regarded as a mere illusion.” Note that this is no error in translating from German, as this book is based on the Shearman lectures delivered in English at University College London, in 1948. Note, furthermore, that while it might be mistaken to speak of illusory appearances in Kantian thought, as we cannot know the noumenal world and thus cannot know whether appearances are illusory, Schopenhauer can speak of the illusory character of representations as Schopenhauer is convinced the thing‐it‐itself (i.e., the will) can be known. Hence, where it might be problematic to speak of *illusory* representations for Kant, as the thing‐in‐itself cannot be known, Schopenhauer can speak of the falsification of representations in light of the thing‐in‐itself, see Berger 2000, e.g., pp. 314–315. Schrödinger wrote about Schopenhauer's conception of the thing‐in‐itself: “I have no doubt that this is our philosopher's eternal, motionless One,” in Schrödinger 1996, on p. 29. I argue the resemblance with Śaṅkara's notion of illusory appearances—where illusory appearances are understood as existing by convention while bearing no ultimate reality—is warranted, as Schrödinger explicitly falsified the plurality we observe in light of the underlying monist unity, which is at the core of his doctrine of identity. Lastly, note that this illusory character did not mean Schrödinger did not take the observed world (including as described by physics) seriously, as I will elaborate on later in this section. Moore noted that Schrödinger “never allowed his distrust of Maya to inhibit his enjoyment of its illusory pleasures. He found happiness in his intellectual work, the companionship of friends, good wine, poetry and drama, the love of women, and explorations of mountains and seashores,” in Moore 1989, on p. 176.

116 When talking about this distinction in reference to Schrödinger, I refer to the perceived‐ultimate distinction rather than the conventional‐ultimate distinction, as Schrödinger did not use these terms.

117 Banks 2021, p. 259.

118 Schrödinger 1964, on p. 37.

119 Schrödinger 1996, on pp. 93–94.

120 Schrödinger 1964, on p. 37.

121 Recall that Schrödinger wrote: “the plurality that we perceive is only an appearance; it is not real.” Ibid., on p. 16.

122 He wrote about this tendency that we exclude ourselves from the world around us that it is a definite feature of our understanding of nature, but that “[…] only a fool would forgo it! Quite right, only a fool.” Schrödinger 1996, on p. 93.

123 Ibid., on pp. 93–94.

124 Ibid., on p. 94.

125 Bitbol understands Schrödinger to maintain “everything happens on the surface of ‘this one thing—mind or world,’” in Bitbol 1996, on p. 67. Note, here, how this understanding of Schrödinger's thought conflicts with current day conceptualizations of Machian neutral monism, where, for Mach: “Consciousness is a set of complex functional relations, not a single unitary phenomenon. The unity of consciousness is an illusion,” in Banks 2021, on p. 260. This analysis of Mach's position seems to contrast directly with Schrödinger's emphasis on an ultimate unitary self or consciousness.

126 Although Michel Bitbol did not mention this, his comments seem to be in line with my more charitable reading. Bitbol wrote that this “real world around us” is “a construct out of Machian elements” and that “this ‘real world around us’ is only a construct […],” in Bitbol 1996, on p. 13—indicating this world around us is a conventional construct with no metaphysical character.

127 Bitbol 1996.

128 Blackburn 1993.

129 Ibid., on p. 15.

130 Bitbol 1996, on p. 13.

131 Ibid., on pp. 39–40.

132 Schrödinger 1996, on p. 95.

133 Bitbol delivered a clear analysis of Schrödinger's understanding of “reality,” in Bitbol 1996, on pp. 12–15.

134 Ibid.

135 Ibid., e.g., on pp. 67 or 140–141.

136 In his 1996 book, Bitbol discusses Indian insights in Schrödinger's thought in relation to Schrödinger's insistence on having a “world‐picture.” Ibid., on p. 67. This emphasis on pictures in Schrödinger's conception of scientific theories is an aspect where we see the influence of Boltzmann on Schrödinger, see Stöltzner 2012.

137 Schrödinger clearly put emphasis on taking these constructs seriously, e.g., in Schrödinger 1964, on p. 64.

138 For Śaṅkara, it is *avidyā* (ignorance) that causes us to superimpose (*adhyāsa*) empirical qualities on spiritual subjectivity. For Schrödinger, the plurality we perceive is “produced by a deception (Indian MAYA).” Schrödinger 1992, on p. 89. In his self‐written Upaniṣad, Schrödinger wrote that we can conquer death through thought, indicating knowledge allows us to conquer death, and that it is a lack of knowledge, or ignorance, that is involved in our perception of a personal (conventional) and finite self. Erwin Schrödinger, Diary 1942–1944 [Ephemerides], 1944, Austrian Central Library for Physics, Vienna/Erwin Schrödinger Archive, L33‐557, copy, in parts transcribed by Auguste Dick.

139 It is important to highlight that neither for Schrödinger nor for Śaṅkara does the conventional‐ultimate distinction entail ontological duplication: ultimately, there is only unity, and the perceived appearances are illusory. Note, here, that Schrödinger also explicitly rejected ontological duplication of the realm of perceptions and the realm of things. Schrödinger wrote: “the story is occurring only once, not twice,” in Schrödinger 1992, on p. 145. Schrödinger's monistic doctrine entailed exactly that there is no ultimate difference between the representation and that which it represents, see Bitbol 1996, on p. 67. Note, then, that this is at least not compatible with a reading of Kant where the phenomenal‐noumenal distinction is ontological (where these are separate words). Note, lastly, that Schrödinger also rejected ontological duplication when it comes to a microscopic and a macroscopic world, see Schrödinger 1935, on pp. 107–119.

140 Both Schopenhauer and Anquetil‐Duperron arrived at a synthesis of Kantian thought and Śaṅkara's Advaita Vedānta. Although their analyses are highly criticized, as discussed earlier, this might indicate Schrödinger adopted a similar synthesis of the two as well. Note, here, that where Schopenhauer believes this noumenal world can be known (i.e., will‐to‐live), and that Schrödinger seems to accept the universal consciousness (often expressed in Śaṅkara's *brahman*) as the ultimate nature of reality.

141 Schrödinger is described as a reluctant revolutionary in Moore 1989, on p. 177, and “prudent” in Bitbol 1998, pp. 3 and 19, in relating his philosophical thought directly to his physics. On this note, I could add that, given Schrödinger's fanatic involvement in philosophy and philosophy for Schrödinger being neither merely a hobby nor an afterthought to his work in physics, an influence of philosophical ideas on Schrödinger's work should not be rejected prematurely, as Lisa Wessels noted in Wessels 1975, on pp. 75–78.
